# Protocol to differentiate glycosylphosphatidylinositol-anchored prion protein from pro-prion protein in cancer cells

**DOI:** 10.1016/j.xpro.2023.102298

**Published:** 2023-06-12

**Authors:** Huan Li, Jie Yang, Jingfeng Li, Zhenxing Gao, Jiang Xu, Chaoyang Li

**Affiliations:** 1 Affiliated Cancer Hospital and Institute of Guangzhou Medical University, State Key Laboratory of Respiratory Disease, Key Laboratory for Cell Homeostasis and Cancer Research of Guangdong High Education Institute, 78 Heng Zhi Gang Road, Guangzhou 510095, China; 2 Department of Stomatology, First Affiliated Hospital, School of Medicine, Shihezi University, No. 107 North 2nd Road, Shihezi, Xinjiang 832008, China

**Keywords:** Antibody, Cancer, Cell Biology, Flow Cytometry/Mass Cytometry, Protein Biochemistry

## Abstract

Defects of glycosylphosphatidylinositol (GPI)-anchor synthesis lead to the production of pro-proteins with altered functions. However, pro-protein-specific antibodies for functional analysis are lacking. Here, we present a protocol to differentiate GPI-anchored prion protein (PrP) from pro-PrP in cancer cells using a complementary approach applicable to other GPI-anchored proteins. We first describe steps for phosphatidylinositol-specific phospholipase C treatment and flow-cytometry-based detection. We then detail the carboxypeptidase Y (CPDY) assay including antibody immobilization, affinity purification, CPDY treatment, and western-blot-based detection.

For complete details on the use and execution of this protocol, please refer to Li et al. (2022).^1^

## Highlights

• A complementary approach to differentiate GPI-anchored PrP from pro-PrP in cancer cells
• Steps to perform PI-PLC treatment and detection by flow cytometry
• Details on CPDY treatment assay and detection by western blot
• Applicable to differentiate other GPI-anchored proteins from their pro-proteins

## Abstract (editor highlights)

Publisher’s note: Undertaking any experimental protocol requires adherence to local institutional guidelines for laboratory safety and ethics.

## Graphical abstract

**Figure:**
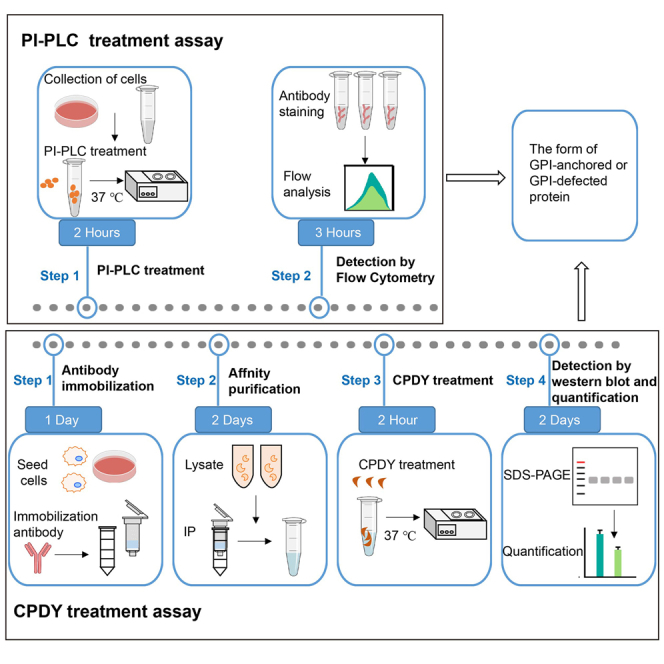

## Before you begin

**CRITICAL:** All materials used for cell culture need to be sterilized, and all procedures for handling cells need to be carried out in a Level II biosafety cabinet under standard aseptic techniques.

***Note:*** This protocol has been successfully used for identifying the expression form of prion protein (PrP) in pancreatic ductal adenocarcinoma cells and melanoma cells.^1^^,^^2^ Here, we describe the detailed steps using hepatocellular carcinoma (HCC) cells as an example. But we expect this will be applicable to other cell lines.

### Cell preparation

**Timing: 1 week**

1. Passage the proliferative human pancreatic cancer cell line AsPC-1 and liver cancer cell line HLE with 0.25% Trypsin-Ethylene Diamine Tetraacetie Acid (EDTA) solution.

***Note:*** Cells need to be split from one 10 cm Perti dish to three 6 cm Petri dish or from one 10 cm Perti dish to two 10 cm Petri dish after 2–3 days of culture. Recommended continuous passage times for cells is less than 10.

2. Seed the cells for further analysis.
  a. For phosphatidylinositol-specific phospholipase C (PI-PLC) treatment.
    i. Plate the cells in a 6 cm Petri dish at a density of 1 × 10^5^ cells.
  b. For carboxypeptidase Y (CPDY) treatment.
    i. Plate the cells in a 10 cm Petri dish at a density of 1 × 10^6^ cells.
3. Culture the cells at 37°C with 5% CO_2_ in a humidified chamber.

***Note:*** HLE cells are cultured in Dulbecco’s modified Eagle’s medium (DMEM) with 10% FBS and 1% Penicillin-Streptomycin (P/S). AsPC-1 cells are cultured in RPMI1640 containing 10% FBS, 1% sodium pyruvate, and 1% P/S.AsPC-1 cells are known to express GPI-anchored PrP.^3^

***Note:*** Warm phosphate buffered saline (PBS) (Table 1) and culture medium to 37°C before starting cell culture.

**Table 1:** Phosphate buffered saline (PBS)

Reagent	Final concentration	Amount
NaCl	137 mM	8 g
KCI	2.7 mM	0.2 g
Na_2_HPO_4_	10 mM	1.44 g
KH_2_PO_4_	1.8 mM	0.24 g
ddH_2_O	N/A	To 1 L
**Total**	**N/A**	**1 L**

Store at room temperature (20°C–25°C). The solution is stable for up to 3 months.

### Reagent preparation

Prepare the specified buffers and solutions as described below.

**Timing: 2–4 h**

4. PI-PLC stock solution
  a. Dissolve PI-PLC to a final concentration of 1 unit/mL in the PI-PLC storage buffer (Table 2). Aliquot and store the stock solution at −20°C, protected from light.

**Table 2:** PI-PLC storage buffer (pH = 7.4)

Reagent	Final concentration	Amount
Tris-HCl	10 mM	8 g
NaCI	144 mM	0.2 g
BSA	0.05%	0.025 g
ddH_2_O	N/A	To 50 mL
**Total**	**N/A**	**50 mL**

Store at room temperature. The solution is stable for up to 3 months.
5. CPDY stock solution
  a. Dissolve CPDY in 10 mM Tris-HCl (pH7.5) (the final concentration is 1 unit/μL). Aliquot and store the stock solution at -20°C, protected from light.

**CRITICAL:** CPDY causes skin and eye irritation. Inhalation may cause allergic or asthmatic symptoms or breathing difficulties. This should be prepared in a fume hood.

## Key resources table

**Table:** 

REAGENT or RESOURCE	SOURCE	IDENTIFIER
**Antibodies**		

8B4	N/A	N/A
4H2	N/A	N/A
Mouse IgG1	BioLegend	Cat# 400165; RRID:AB_11150399
Alexa Fluor 488 nm conjugated goat anti-mouse IgG	Thermo Fisher Scientific	Cat# A-11001; RRID:AB_2534069

**Chemicals, peptides, and recombinant proteins**		

0.25% Trypsin-EDTA solution	Gibco	Cat#25200056
RPMI1640	Gibco	Cat# C11875500BT
Sodium pyruvate	Thermo Fisher	Cat#11360070
Cell scraper	BKMAM	Cat#BKM-002
cOmplete™ Proteinase inhibitor cocktail	Roche	Cat#11697498001
Phenylmethanesulfonyl fluoride (PMSF)	Sigma	Cat# 93482
Dulbecco’s Modified Eagle’s Medium (DMEM)	Thermo Fisher	Cat#C11995500BT
Phosphatidylinositol specific phospholipase C (PI-PLC)	Sigma	Cat#P5542
Nitrocellulose membranes	Merck Millipore	Cat#HATF00010
Aminolink plus Coupling Resin	Thermo Fisher	Cat#20501
Pierce Spin Cups (spin column and collecting tube)	Thermo Fisher	Cat#69700
Carboxypeptidase Y (CPDY)	Sigma	Cat#9046-67-7
Fetal bovine serum (FBS)	Gibco	Cat# 10099141C
Penicillin-streptomycin	Biological Industries	Cat#03-031-1
Tris-base	BioFROXX	Cat#1115GR500
Sodium chloride (NaCI)	Sinopharm Chemical	Cat#10019318
Sodium dihydrogen phosphate (Na_2_HPO_4_)	Sinopharm Chemical	Cat#20040618
Ethylenediaminetetraacetate (EDTA)	Sigma	Cat#V900106
Ethylene glycol tetraacetic acid (EGTA)	Sigma	Cat#324626
Sodium pyrophosphate	Macklin	Cat#S817837
Sodium hydroxide (NaOH)	Sinopharm	Cat#10019764
Sodium cyanoborohydride	Aladdin	Cat# S432484
hydrochloric acid (HCI)	Sinopharm	Cat# 02-10011061
β-glycerophosphate	Sigma	Cat#G9422
Sodium orthovanadate (Na_3_VO_4_)	Sigma	Cat#S6508
Triton X-100	Sigma	Cat#T8787
Sodium dodecyl sulfate (SDS)	Sigma	Cat#V900859
2-mercaptoethanol	Sigma	Cat#M3148
Glycerol	Macklin	Cat#G810575
Bromophenol blue	Life Science Production	Cat#0449
Potassium chloride (KCI)	Sinopharm Chemical	Cat#10016318
Potassium dihydrogen phosphate (KH_2_PO_4_)	Sinopharm Chemical	Cat#10017618
Bovine serum albumin (BSA)	MP Biomedicals	Cat#FC0077
Glycine	BioFROXX	Cat#1275GR500
N, N, N′, N′-tetramethylethylenediamine (TEMED)	Macklin	Cat#T6023
Ammonium persulfate (APS)	Aladdin	Cat#A112450
ProClean 300	Beyotime	Cat# ST853
BCA protein assay kit	Thermo Fisher	Cat#23227
Methanol	Sinopharm	Cat#H1J0106
Immobilon western chemilum HRP substrate	Merck Millipore	WBKLS0500
Tween-20	BioFROXX	Cat#1247ML100

**Experimental models: Cell lines**		

AsPC-1	ATCC	Cat# CRL-1682
HLE	IMMOCELL	Cat# IM-H278

**Software and algorithms**		

Prism 8	GraphPad	https://www.graphpad.com/
Flowjo 7.6	FlowJo LLC	www.flowjo.com
ImageJ^4^	FIJI	https://fiji.sc

**Other**		

Wet-blot transfer equipment	Bio-Rad	Cat#1703930
BD symphony cytometer	Becton, Dickinson and Company	Cat# FACSTM C6
100 mesh gauze net	LKBIO	Cat#DHLH-SW10
FACS tubes	Corning	Cat#352058
Milli-Q water purification system	Millipore	Cat#C85358
Sonicator with tip probe	Fisher Scientific	Cat#FB505
Water bath (at 37°C)	Shanghai Jinghong Technical	Cat#XMTD-8222
Platform shaker	Kylin-Bell	Cat#VORTEX-6
Multipurpose Shaker	Kylin-Bell	Cat#QB-206
Cell culture incubator with temperature and CO_2_ control	Thermo Scientific	Cat#SERIES 8000
Automated cell counter	Nexcelom	Cat#Cellometer Auto 1000
Chemiluminescence gel imager	Tanon	Cat#Tanon 4800
Cell counting slides	Nexcelom	Cat#CHT4-SD100-514
Gel casting and electrophoresis equipment	Bio-Rad	Cat# 1658001
Power supply for gel electrophoresis and blotting	Bio-Rad	Cat# 1645050
1.5 mL Microcentrifuge tubes	Axygen	Cat#MCT-150-C
15 mL Centrifugal tube	NEST	Cat#601052
Cell culture dishes, 6 cm, round	NEST	Cat#705001
Cell culture dishes, 10 cm, round	NEST	Cat#704002

## Materials and equipment

***Note:*** Add 1× complete™ the proteinase inhibitor cocktail and 1mM phenylmethanesulfonyl fluoride (PMSF) before use.

**CRITICAL:** SDS causes respiratory tract irritation, it should be weighed in the fume hood. Wear personal protective equipment.

• Washing buffer: 5.84 g NaCI in 100 mL ddH_2_O.

***Note:*** Store at 4°C for up to 3 months.

• 1 U/mL PI-PLC: 5 U PI-PLC in 5 mL PI-PLC Storage buffer

***Note:*** Store at -20°C for up to 2 years.

• 1 U/μL CPDY: 1 mg CPDY (50 unit) in 50 μL 10 mM Tris-HCl (pH7.5).

***Note:*** Store at -20°C for up to 3 months.

• Sodium cyanoborohydride solution (5 M): 0.013 g Sodium cyanoborohydride in 40 μL NaOH (1 M).

***Note:*** Use freshly prepared solution, sodium cyanoborohydride is toxic. Wear gloves when handling.

• 100 mM PMSF: 1.74 g PMSF in 100 mL isopropanol.
• 50× cOmplete™ proteinase inhibitor cocktail: a pill of medicine in 1 mL cell lysis buffer (Table 3).

**Table 3:** Cell lysis buffer

Reagent	Final concentration	Amount
Tris-HCI pH 7.5 (1 M)	20 mM	10 mL
NaCI (3 M)	150 mM	25 mL
Na_2_HPO_4_	10 mM	1.44 g
EDTA (0.5 M)	1 mM	1 mL
Ethylene Glycol Tetraacetic Acid (EGTA) (0.5 M)	1 mM	1 mL
Sodium pyrophosphate (250 mM)	2.5 mM	5 mL
β-glycerophosphate (100 mM)	1 mM	1 mL
Na_3_VO_4_ (100 mM)	1 mM	5 mL
Triton X-100	1% (V/V)	5 mL
ddH_2_O	N/A	To 500 mL
**Total**	**N/A**	**500 mL**

Store at 4°C for up to 1 year.

***Note:*** Store at −20°C for up to 3 months.

• TBST: add 1 mL Tween 20 in 1 L 1× TBS. To make 1× TBS: add 100 mL 10× TBS (Table 4) in 900 mL ddH_2_O.

**Table 4:** 10× Tris buffered saline (TBS)(5L)

Reagent	Final concentration	Amount
Tris-base	20 mM	61 g
NaCI	150 mM	438.75 g
ddH_2_O	N/A	To 5 L
**Total**	**N/A**	**5 L**

Store at room temperature. The solution is stable for up to 3 months.

***Note:*** Store at room temperature for up to 3 months.

• To make fresh 1× transfer buffer (1 L): add 100 mL 10× transfer buffer (Table 5), 150 mL methanol in 750 mL ddH_2_O. To make 1 × running buffer (1 L) : add 100 mL 10× running buffer (Table 6) in 900 mL ddH_2_O.

**Table 5:** 10× Transfer buffer without methanol

Reagent	Final concentration	Amount
Tris-base	250 mM	30.3 g
Glycine	1.92 M	144.2 g
ddH_2_O	N/A	To 1 L
**Total**	**N/A**	**1 L**

Store at room temperature. The solution is stable for up to 3 months.

**Table 6:** 10× Running buffer

Reagent	Final concentration	Amount
Tris-base	250 mM	30.3 g
Glycine	1.92 M	144.2 g
SDS	1 %	10 g
ddH_2_O	N/A	To 1 L
**Total**	**N/A**	**1 L**

Store at room temperature. The solution is stable for up to 3 months.

**CRITICAL:** The HCl fume is toxic and causes respiratory track and skin irritation. Open the bottle containing HCl in the fume hood. Wear personal protective equipment.

## Step-by-step method details

We describe two assays that are performed independently. For cell surface PI-PLC treatment assay, please refer to steps 1–15. For CPDY treatment assay, please refer to steps 16–48.

### Cell surface PI-PLC treatment assay

#### Collection of cells

**Timing: 30 min; day 1**

This procedure details how to prepare and collect cells.

1. Collect cells.
  a. Seeding 5 × 10^5^ cells in a 6 cm Petri dish 12–24 h before experiment. Once cell number reaches 1 × 10^6^, aspirate off the cell culture medium and discard.
  b. Wash the three 6 cm Petri dishes once with 2 mL PBS for each Petri dish and discard the PBS.
  c. Add 1 mL of 0.05% trypsin-EDTA into each Petri dish for 2 min at 37°C in a humidified chamber.
  d. Add 3 mL of cell culture medium into each Petri dish to stop the cell digestion and transfer the cells with a pipette into a 15 mL centrifuge tube.
  e. Centrifuge the tubes at 300 × *g* for 3 min at room temperature.
2. Discard the supernatant, resuspend the cells of three 15 mL centrifuge tube with 1 mL ice-cold PBS, and transfer the cells to three 1.5 mL microcentrifuge tubes.

### *PI-PLC treatment assay*

**Timing: 1 h; day 1**

The following steps detail how to treat cells with PI-PLC.

3. Wash the cells of three microcentrifuge tubes twice with 1 mL ice-cold PBS by centrifuging at 300 × *g* at 4°C for 3 min.
4. Add 200 μL (0.1 U/mL) PI-PLC into one of the three microcentrifuge tubes and add 200 μL of PBS into the two remaining microcentrifuge tubes, label the tubes for PI-PLC treatment or no PI-PLC treatment, and resuspend the cells with gentle pipetting (essential)

***Note:*** PI-PLC enzymatic activity is not affected by the presence of small amount of divalent metal ions. 1 U PI-PLC can be diluted with ice-cold PBS. Diluted PI-PLC can be stored at -20°C.

5. Incubate the microcentrifuge tubes at 37°C for 1 h and gently flick the tubes every 10 min (essential)
6. Centrifuge the tubes at 300 × *g* for 3 min and discard the supernatant.
7. Wash the cells once with 1 mL of PBS and centrifuge the tubes at 300 × *g* for 3 min.

***Note:*** From this step on, a common problem frequently encountered is the loss of cells, be careful to handle the cells.

### *Flow cytometry assay: Staining*

**Timing: 1.5 h; day 1**

This procedure details how to stain cells with indicated antibody for flow cytometry and/or immunofluorescence staining analysis.

8. Discard the supernatant and add antibodies to the tubes.
  a. Add 200 μL of PBS containing the antibodies (mouse monoclonal antibody 4H2 against PrP) at the final concentration of 10 ng/μL^5^ into the tube with PI-PLC treatment.
  b. Add 200 μL of PBS containing the antibodies (mouse monoclonal antibody 4H2 against PrP) at the final concentration of 10 ng/μL into another tube without PI-PLC treatment.
  c. Add 200 μL of PBS containing isotype control mouse IgG1 (final concentration is 10 ng/μL) into the other tube without PI-PLC treatment and vortex briefly.
  d. Incubate the antibody-cell mixture at 4°C for 40 min (essential)
9. Wash the antibody-cell mixture with 1 mL of ice-cold PBS, vortex the tubes briefly and centrifuge at 300 × *g* for 3 min at 4°C. Discard the supernatant. Repeat process 5 more times (essential)

***Note:*** Carefully aspirate off the wash solution to avoid loss of cells.

10. After final centrifugation, discard the supernatant and add antibodies to the tubes.
  a. Add 200 μL of PBS containing fluorescent dye conjugated secondary antibody (goat- anti-mouse AF488 at a final concentration of 5 ng/μL), vortex briefly.
  b. Incubate at 4°C for 40 min in the dark (essential).
11. As before wash the antibody-cell mixture 6 times with 1 mL of ice-cold PBS each time, vortex briefly, and centrifuge at 300 × *g* for 3 min at 4°C.
12. Discard the supernatant each time and resuspend the cells with 200 μL ice-cold PBS at the end.

***Note:*** The suspension volume can be scaled-up or -down according to the cell numbers. We normally keep the final volume at 200 μL.

13. Filter the cells with 100 mesh gauze net, and collect the cells in pre-labelled FACS tubes.

### *Flow cytomery assay: Data collection*

**Timing: 30 min**

This procedure details how to collect data on a BD symphony cytometer (FACSTM C6). Any flow cytometer with similar functions can also be used.

**Figure 1:**
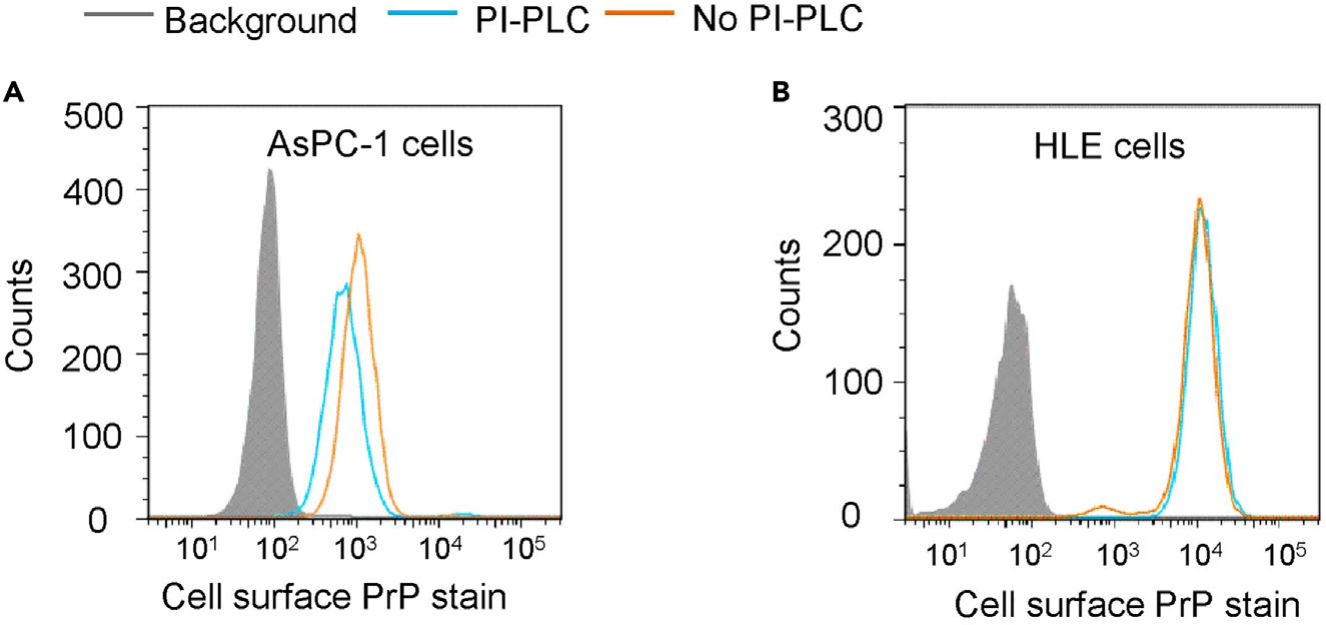
Flow cytometry assays showed that PrP on the surface of HLE cells was resistant to PI-PLC treatment Cell surface PrP was stained with 4H2 with or without PI-PLC treatment (as shown in this figure, blue or brown line, respectively). (A) PrP on the surface of AsPC-1 cell was sensitive to the treatment. (B) HLE cell surface PrP was resistant to the treatment. Background stain was shown as shaded region: cells stained with mouse IgG1.

14. Collect data on a BD Symphony flow cytometer and analyze by FlowJo software.
  ***Note:*** The cells are gated based on their light-scattering properties and fluorescent intensities from antibody staining, followed by analysis. Samples without PI-PLC treatment but incubated with IgG1 or 4H2 are used to set up voltages defining the negative and positive staining signals.
  ***Note:*** Single fluorescence dying does not need to adjust compensation.
  a. Inset the FACS tube for IgG1 staining, select the drawing tool in the screen to draw one Dot Plot of omnidirectional and lateral angles. X-axis label is FSC-A, Y-axis label is SSC-A. Set appropriate photomultiplier Tube (PMT) voltage. Painting single cell gating to analyze cell population.
  b. Draw a histogram with the cells in the selected gate. Set fluorescence staining value as X-axis. Set Y-axis as number of cells counted. The fluorescence staining value of IgG1 stained sample is set to start from 0. The number of cells collected is 10,000.
  c. After finish IgG1 sample, replace the IgG1 tube with the 4H2 tubes treated or not treated with PI-PLC with the same setting. Draw histograms for these two samples with the same selected gate.
  d. Record the samples one at a time.
15. Analyze the results with FlowJo (Figure 1).

### CPDY treatment assay

#### Antibody immobilization

**Timing: 2 h; day 1**

This step details how to generate antibody conjugated beads.

**Figure 2:**
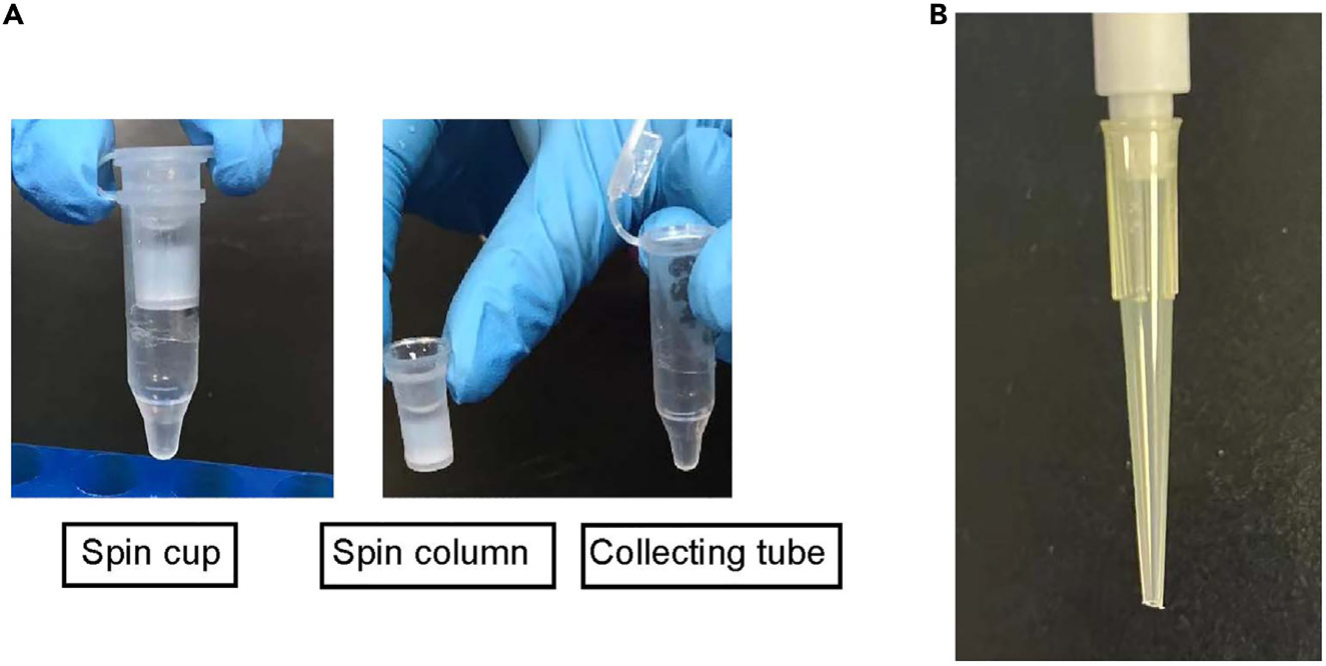
The picture of a spin cup and a pipette with tip-cut (A) The picture of a spin cup. (B) The picture of a pipette with tip-cut.

***Note:*** To ensure the amount of intact resins in all columns is equal, the 200 μL pipette tip that is used to transfer the beads is cut off with scissors at 5 millimeter from the tip (Figure 2B). Shake the beads solution well each time and transfer the equilibrated resins into the columns.

16. Equilibrate the AminoLink Plus Coupling Resin and reagents (stored in 4°C fridge) to room temperature.
17. Resuspend the AminoLink Plus Coupling Resin well with gentle tapping, transfer 100 μL of beads (50% slurry) to a spin column (Pierce Spin Cups, Upper spin column). Place the column in a clean 1.5 mL microcentrifuge tube (Pierce Spin Cups, lower tube) (Figure 2A).

18. Centrifuge the columns at 1,000 × *g* for 1 min at room temperature and discard the flow-through.
19. Wash the beads twice with 300 μL binding buffer (Table 7), centrifuge at 1,000 × *g* for 1 min, and discard the flow-through.

**Table 7:** Binding buffer (pH = 7.2)

Reagent	Final concentration	Amount
Sodium phosphate	0.5 mM	0.008 g
NaCI	7.5 mM	0.043 g
HCI	N/A	Adjust pH to 7.2
ddH_2_O	N/A	To 100 mL
**Total**	**N/A**	**100 mL**

Store at 4°C for up to 3 months.
20. Add 100 μg of 8B4 antibody and adjust the volume to 300 μL with binding buffer (Table 7) (essential)

***Note:*** Antibodies have different affinity and storage conditions. Consult the relevant information for affinity purification experiments.

***Note:*** Make sure that the antibody solution does not contain reducing reagent as it will greatly affect the efficiency of antibody coupling.

21. Add 4.5 μL of 5 M sodium cyanoborohydride solution into the antibody-beads column (essential).
22. Rotate the column for 120 min at room temperature.
23. Centrifuge the column at 1,000 × *g* for 1 min at room temperature and save the flow-through to check the efficacy of antibody conjugating.

***Note:*** Using bicinchonininc acid (BCA) protein assay kit to determine antibody concentration in the flow-through to check the efficiency of antibody coupling before step 24.

24. Wash the beads twice with 300 μL of binding buffer (Table 7) each time, centrifuge and discard the flow-through.
25. Wash the beads once with 300 μL of tris buffer (Table 8), centrifuge and discard the flow-through.

**Table 8:** Tris buffer (pH)

Reagent	Final concentration	Amount
Tris-base	1 M	12.11 g
HCI	N/A	adjust pH as specified
ddH_2_O	N/A	To 100 mL
**Total**	**N/A**	**100 mL**

Store at 4°C for up to 3 months.
26. Add 300 μL of tris buffer (Table 8) to the column and 4.5 μL of the sodium cyanoborohydride solution in a fume hood, rotate the column for 15 min at room temperature.
27. Centrifuge the column at 1,000 × *g* for 1 min at room temperature and discard the flow-through.
28. Wash the antibody-bead mixture six times with 200 μL of washing buffer each time, centrifuge at 1,000 × *g* for 1 min and discard the flow-through.
29. If the column is not used right away, wash the beads twice with 300 μL of binding buffer (Table 7), centrifuge at 1,000 × *g* for 1 min and discard the flow-through.
30. Store the resin in binding buffer (Table 7) containing 0.1% Antibacterial and preservative agents (ProClean 300) at 4°C.

***Note:*** Add antibacterial and preservative agents (ProClean 300) during storage.

### *Making cell lysates*

**Timing: 1 h; day 1**

This procedure details how to prepare cell lysate for affinity purification.

31. 24 h before making cell lysate, seed 5 × 10^6^ cells in a 10 cm Petri dish. Discard cell culture medium and wash the cells gently three times with ice-cold PBS by pipetting along the edge of the Petri dish. Discard the PBS after each wash.
32. Add 500 μL ice-cold cell lysis buffer (Table 3) to the cells, scrape the cells and incubate the cell-lysate at 4°C for 5 min.

***Note:*** Cool down the lysis buffer and PBS at 4°C. Cool down the table-top centrifuge to 4°C before step 31. Add protease and phosphatase inhibitors to cell lysis buffer just before cell lysis.

33. Transfer the lysate to a 1.5 mL tube and sonicate the cell lysate with an ultrasonic cell pulverizer (Scientz-11D, Ningbo Scientz Biotechnology CO.LTD, China) for 1 min at 120 Watts (Ultrasonic 5 s/ interval 5 s).

***Note:*** Adjust the ultrasonic condition to reduce the generation of foam and splashes. Sonicate 2 minutes at 60 Watts to produce homogenous cell lysates. The ultrasonic horn must be inserted into the sample before starting; the probe should be centered and not attached to the tube; the ultrasonic time should not exceed 5 s each time, and the interval time should be greater than or equal to the ultrasonic time.

34. Place the tube on ice for 20 min and then centrifuge the tube at 13,000 × *g* for 10 min at 4°C to get rid of non-dissolved cell debris.

***Note:*** Cell collection, sonication, and the long-term incubations are performed at 4°C.

***Note:*** To decrease nonspecific binding, the cell lysates can be pre-cleaned with 80 μL agarose beads. Incubate the agarose beads with the cell lysate on a rotator for 30 min to 1 h at 4°C. Spin down the beads at 1,000 × *g* for 1 min and collect the supernatant.

### *Affinity purification of PrP*

**Timing: 12 h; day 1**

This procedure details the purification of PrP in cells.

35. Transfer the supernatant to a spin column containing the aminolinked antibody (volume: about 500 μL) for affinity purification.
36. Incubate the column on a rotator overnight at 4°C (at least 12 h).
37. Centrifuge the column at 1,000 × *g* for 1 min at 4°C and discard the flow-through.
38. Wash the beads six times with 300 μL lysis buffer (Table 3) by turning the column upside down 10 times, and centrifuge the column at 1,000 × *g* for 1 min at 4°C. Discard flow-though.

***Note:*** We do recommend washing the beads with lysis buffer (Table 3) but not with PBS or other buffers during step 38.

39. Add 10 μL of neutralization buffer (Table 9) to the collection tube, add 20 μL of elution buffer (Table 10) in the column and incubate for 5 s, centrifuge the column at 1,000 × *g* for 1 min at 4°C (essential)

**Table 9:** Neutralization buffer (pH = 9.5)

Reagent	Final concentration	Amount
Tris-base	1 M	60.57 g
NaCI	1.5 M	43.83 g
EDTA	1 mM	0.15 g
ddH_2_O	N/A	To 500 mL
**Total**	**N/A**	**500 mL**

Store at 4°C for up to 3 months.

**Table 10:** Elution buffer (pH = 2.8)

Reagent	Final concentration	Amount
Glycine	50 mM	3.75 g
HCI	N/A	Adjust pH to 2.8
ddH_2_O	N/a	To 500 mL
**Total**	**N/a**	**500 mL**

Store at 4°C for up to 3 months.
40. Add 100 μL of elution buffer (Table 10) to the column and incubate for 15 min at 4°C. The column does not need to be mixed (essential)
41. Centrifuge the column at 1,000 × *g* for 1 min at 4°C to collect the eluent.

***Note:*** In case the eluent is not enough for carboxypeptidase treatment, repeat affinity purification 2–3 times to collect more eluent.

***Note:*** In case the amount of antibody available for PrP purification is 10–75 μg, the affinity purification step shall be repeated 2–3 times to get enough purified PrP for CPDY treatment (Please see following).

### *CPDY treatment assay*

**Timing: 12 h; day 2**

This procedure details how to perform the CPDY treatment.

42. Prepare four 1.5 mL microcentrifuge tubes. Label the tubes as following: CPDY treatment 0, 30, 60 and 120 min. Add 20 μL sample from step 41 to each tube.
43. Add 0.5 U CPDY to each tube and incubate for indicated periods at 37°C(essential).

***Note:*** It is better to prepare fresh CPDY or use the aliquoted CPDY stored at −20°C, and the amount of CPDY can be adjusted according to the amount of eluent.

44. Add 7 μL 4× SDS Laemmli buffer (Table 11) to each tube once the reaction is done and heat the tube at 100°C for 10 min, then place the tube on ice for at least 5 min.

**Table 11:** 4× SDS Laemmli buffer

Reagent	Final concentration	Amount
Sodium dodecyl sulfate (SDS)	4%	4 g
2-mercaptoethanol	10% (V/V)	10 mL
Tris-HCl pH 6.8 (1M)	125 mM	12.5 mL
Glycerol	20% (V/V)	20 mL
Bromophenol blue	0.004%	4 mg
ddH_2_O	N/a	To 100 mL
**Total**	**N/a**	**100 mL**

**Figure 3:**
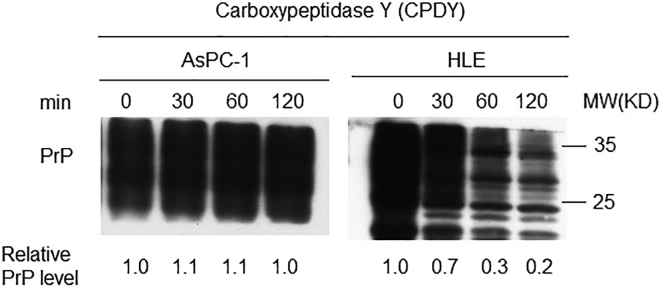
Treatment of PrP purified from HLE cells with CPDY using PrP from AsPC-1 cells as control PrP purified from HLE cell lines by 8B4 from step 41 was quantified first by BCA method and subjected to CPDY treatment as indicated and immunoblotted with 4H2. PrP level was quantified by IMAGE J. The level of PrP without CPDY treatment (time 0) was arbitrarily defined as 1.0. Relative PrP level was calculated as PrP level at specified time-point divided by PrP level at time 0.

***Note:*** PrP molecules can be separated by 10% SDS-PAGE (Table 12).

Store at ≤−20°C for up to 1 year.
45. Analyze 10 μL of sample by western blot (Figure 3).
  a. Prepare a 10% resolving polyacrylamide gels with 5% stacking gel.
  b. Install the gel in the gel running cassette (BioRad, 1658001) and fill in with 1× running buffer. load the samples and the molecular weight marker. Gel running conditions: set the Voltage at 80 V and run for 30 min. Once the proteins enter the resolving gel, re-set the voltage to 120 V and run the gel for 1 h.
  c. Transfer separated proteins to 0.45 μm nitrocellulose (NC) membrane (Merck Millipore, USA) as following:
    i. Soak NC membranes, sponges, and thickened filter paper in transfer buffer for few seconds.
    ii. Remove the gel from the running apparatus and prepare the transfer sandwich by building the following layers in the transfer holder: sponge - thickened filter paper - gel -NC membrane - thickened filter paper- sponge.
    iii. Roll a 25 mL glass centrifuge tube over the NC membrane to get rid of air bubbles trapped between gel and the NC membrane.
  d. Lock the transfer holder and place it into a transfer apparatus. Fill the transfer apparatus with transfer buffer. Conditions of transfer: set current at 0.12 mA (A piece of polyacrylamide gel), transfer for 1 h 30 min. Proteins will migrate from the cathode (-) to the anode (+).
  e. After transfer, block the NC membrane with 3% bovine serum albumin (BSA) in TBST at room temperature for 2 h. After blocking, discard the block solution.
  f. NC membrane is then incubated with 4H2 (final concentration is 1 ng/μL) on a rocker at 4°C overnight. After primary antibody reaction, discard the solution.
  g. Wash the NC membrane three times with TBST for 10 min each time at room temperature and discard TBST.
  h. Add HRP-conjugated secondary antibody (goat-anti-mouse, final concentration is 0.2 ng/μL) and incubate for 1 h at room temperature. After secondary antibody reaction, discard the solution.
  i. Wash the NC membrane three times with TBST for 10 min each time at room temperature and discard the TBST.
  j. Detect chemiluminescence reaction with ECL substrate using a chemiluminescence imager (Tanon 4800).

**Table 12:** 10% SDS-PAGE

Reagent	Resolving solution	Stacking solution
ddH_2_O (mL)	2.87	3.515
30% acrylamide (mL)	3.33	0.835
Tris-HCl pH 8.8 (mL)	3.73	0
Tris-HCl pH 6.8 (mL)	0	0.625
10% SDS (mL)	0.1	0.05
10% ammonium persulfate (APS) (μL)	50	25
TEMED (μL)	10	5
**Total**	**10 mL**	**5 mL**

Store the gel at 4°C for up to 3 days.

### *Used resin storage*

**Timing: 1 h; day 2**

This procedure details how to store the used spin column.

***Note:*** To save time and expense, antibody conjugated resin can be re-used, the used spin column is stored in a 4°C refrigerator.

46. Add 200 μL of binding buffer (Table 7) to the column, centrifuge at 1,000 × *g* for 1 min and discard the flow-through.
47. Repeat this step once.
48. Add 200 μL of binding buffer (Table 7) to the column. For long-term storage, add 0.1% Antibacterial and preservative agents (ProClean 300).

***Note:*** The used spin column can be re-used for about 3 times. Seal the lid with sterile parafilm.

## Expected outcomes

The PrP in HLE cell lines is pro-PrP. (1) the PrP on the cell surface of HLE cell lines is resistant to PI-PLC treatment (Figure 1). (2) whereas GPI-anchored proteins are resistant to carboxypeptidase Y treatment,^6^ the PrP purified from HLE cell lines is more sensitive to CPDY treatment in a time-dependent manner than PrP from AsPC-1 (Figure 3). After treatment with the enzyme for 120 min, the amount of purified PrP from HLE cells is reduced by more than 80% (Figure 3).

## Quantification and statistical analysis

Data were quantified using densitometry with ImageJ (https://fiji.sc). Flow cytometry results were analyzed using FlowJo 7.6 (www.flowjo.com).

## Limitations

Although most GPI-anchored proteins can be cleaved by PI-PLC on cell surface, there are GPI-anchored proteins resistant to PI-PLC digestion due to the presence of an additional acyl chain, which can’t be cut by PGAP1. Two approaches can be adopted. First, treating the purified protein with carboxypeptidases which cleave proteins from the carboxyl-terminus if it is not protected by GPI-anchor. This approach requires the target protein to be purified. However, purification of a protein could be a daunting project by itself in the absence of a good amount of high-quality antibody. An alternate approach is to check the expression level of PGAP1 and to over-express PGAP1, then perform PI-PLC treatment to check the effect on the target protein.

## Troubleshooting

### Problem 1

Double peaks appear in the PI-PLC treated or not treated cells (PI-PLC assay-Date Collection, step 15).

### Potential solution

• Increase the amount of primary antibody and/or lengthen the incubation time of primary antibody.
• Shake the antibody-cell mixture every 10 min. Make sure all the steps were performed at 4°C.

### Problem 2

No peak shift under PI-PLC treatment in positive control group (PI-PLC assay-Date Collection, step 15).

### Potential solution

• PI-PLC is not working, use freshly made PI-PLC solution.
• Antibody may lose efficacy during storage or re-usage, try a new batch of antibody. In some cases, the protein epitope may be lost causing the monoclonal antibody not recognizing the target, use another monoclonal antibody targeting another epitope.
• Increase the incubation time of PI-PLC treatment for an additional 30 min, and flick the tubes five times with fingers for every 10 min.
• Reduce cells by one-third to increase the efficiency of PI-PLC cleavage.

### Problem 3

Loss of cells during the washing steps in PI-PLC treatment assay. (PI-PLC assay-Flow cytometry: staining, steps 9 & 11).

### Potential solution

• Centrifuge at 660 × *g* for 3 min, and reduce washing from 6 times to 3 times.
• Vortex the tubes instead of pipetting up-and-down.

### Problem 4

The amount of antibody used for affinity purification is relatively big and the cost is high (Antibody immobilization, step 20).

### Potential solution

• The amount of antibody can be reduced from 100 μg to 10–75 μg. In this case, purification must be repeated as stated in the second note of step 41.

### Problem 5

Antibody is not linked to the resin (Antibody immobilization, step 23).

### Potential solution

• Dialyze the antibody extensively against 1× PBS to get rid of glycine during antibody purification process.
• Buffers should not contain reducing agents (for example: DL-dithiothreitol (DTT) or β-mercaptoethanol) as they can affect the integrity of the antibody.

### Problem 6

Target protein is not purified from sample (CPDY treatment assays-affinity purification steps, step 41).

### Potential solution

• Re-make antibody-resin, use freshly made sodium cyanoborohydride solution.
• Use a different batch of antibody.
• The expression level of a bait protein is too low, repeat steps 35–36 with cell lysates as much as possible or alternatively, incubate a large volume of cell lysate with the antibody conjugated resins in a 10–15 mL tube overnight at 4°C. After this step, transfer the cell lysate-resins back to the spin column at step 35, centrifuge at 1,000 × *g* for 1 min to collect the resins. Repeat the steps as necessary to collect all the resins before step 37.

### Problem 7

Resin leaks from the spin column (CPDY treatment assays, step 48).

### Potential solution

• Use a new column.
• When centrifuging columns, the collection column should not contain excess liquid to reduce back pressure.

## Resource availability

### Lead contact

Further information and requests for resources and reagents should be directed to and will be fulfilled by the lead contact, Chaoyang Li (chaoyangli@gzhmu.edu.cn).

### Materials availability

All reagents generated in this study are available from the lead contacts upon completing a Materials Transfer Agreement.

## Data Availability

This study did not generate and analyze any original datasets/codes.
